# A Novel Mutual Information Based Feature Set for Drivers’ Mental Workload Evaluation Using Machine Learning

**DOI:** 10.3390/brainsci10080551

**Published:** 2020-08-13

**Authors:** Mir Riyanul Islam, Shaibal Barua, Mobyen Uddin Ahmed, Shahina Begum, Pietro Aricò, Gianluca Borghini, Gianluca Di Flumeri

**Affiliations:** 1 School of Innovation, Design and Engineering, Mälardalen University, Högskoleplan 1, 722 20 Västerås, Sweden; shaibal.barua@mdh.se (S.B.); mobyen.ahmed@mdh.se (M.U.A.); shahina.begum@mdh.se (S.B.); 2 BrainSigns srl, Lungotevere Michelangelo 9, 00192 Rome, Italy; pietro.arico@uniroma1.it (P.A.); gianluca.borghini@uniroma1.it (G.B.); gianluca.diflumeri@uniroma1.it (G.D.F.); 3 Department of Molecular Medicine, Sapienza University of Rome, Piazzale Aldo Moro 5, 00185 Rome, Italy

**Keywords:** electroencephalography, feature extraction, machine learning, mental workload, mutual information, vehicular signal

## Abstract

Analysis of physiological signals, electroencephalography more specifically, is considered a very promising technique to obtain objective measures for mental workload evaluation, however, it requires a complex apparatus to record, and thus, with poor usability in monitoring in-vehicle drivers’ mental workload. This study proposes a methodology of constructing a novel mutual information-based feature set from the fusion of electroencephalography and vehicular signals acquired through a real driving experiment and deployed in evaluating drivers’ mental workload. Mutual information of electroencephalography and vehicular signals were used as the prime factor for the fusion of features. In order to assess the reliability of the developed feature set mental workload score prediction, classification and event classification tasks were performed using different machine learning models. Moreover, features extracted from electroencephalography were used to compare the performance. In the prediction of mental workload score, expert-defined scores were used as the target values. For classification tasks, true labels were set from contextual information of the experiment. An extensive evaluation of every prediction tasks was carried out using different validation methods. In predicting the mental workload score from the proposed feature set lowest mean absolute error was 0.09 and for classifying mental workload highest accuracy was 94%. According to the outcome of the study, it can be stated that the novel mutual information based features developed through the proposed approach can be employed to classify and monitor in-vehicle drivers’ mental workload.

## 1. Introduction

Driving is a dynamic and complex set of synchronous actions including various secondary tasks i.e., simultaneous cognitive, spatial and visual tasks. The rapid increase of in-vehicle systems like telematics and infotainment systems increase the number of secondary tasks with the primary task of driving. Along with the workload of natural driving, secondary tasks and different road environments increase the Mental Workload (MWL) of drivers. However, an excessive in-vehicle drivers’ MWL, eventually causing mental fatigue if prolonged over time, can lead to significantly deteriorated driving performance and makes the driver more vulnerable to making mistakes [1,2]. A study revealed that 72% of all the road accidents happen each year due to driver errors [3]. The overwhelming increase in traffic fatalities due to elevated MWL forces the need of determining in-vehicle drivers’ MWL efficiently. Researchers of diverse domains have identified drivers’ MWL assessment mechanisms both in simulated and real environments [2,4,5]. Physiological measures, particularly Electroencephalography (EEG), have been shown to be a suitable measure of MWL [6,7,8]. On the other hand, the process of acquiring EEG signals during natural driving requires complex equipment to be used in addition to the in-vehicle systems. As a result, the process of in-vehicle recording of EEG is not favorable to natural driving. At this point an approach for drivers’ MWL monitoring that contains minimal utilization of EEG signal is a sine qua non.

Several studies have exploited the vehicular parameters such as lateral speed, steering wheel angle, lane change, etc. as a complementary measure to EEG to obtain insight about driver’s psycho-physiological state [9,10]. Also, vehicular parameters are not obstructive during driving in comparison to EEG recording. Therefore, it would be possible to (i) utilize the association of vehicular parameters and EEG signals in terms of Mutual Information (MI) [11] in developing a feature template establishing the combined effect on MWL, and then (ii) this feature template can be further used to evaluate in-vehicle driver’s MWL from the vehicular parameters, which can be easily extracted from the built-in systems of a vehicle. More specifically, the conceived application is to record EEG signals once for a specific driver and a specific vehicle along with different vehicular features while driving, taking advantage of the added value of neurophysiological data (i.e., EEG). A feature template will be created combining the underlying characteristics of EEG and vehicular signals and thus enhancing the statistical power of prediction models. Then, this feature template will be fed with only vehicular data afterwards to generate online assessment of in-vehicle MWL of the driver, thus avoiding repeated use of invasive devices for recording EEG signals in vehicle and performing complex computations as well.

In this context, this study further investigated the possible association between vehicular and EEG signals and their relationship with the MWL of drivers while driving. In particular, the present work validates the fusion of mentioned signals with the aim to develop feature set that can be used for in-vehicle drivers’ MWL evaluation with a provision for reducing the complexity of recording EEG signals repeatedly in the concerned tasks. The aim of this study can be outlined as:

- Develop a new feature fusion methodology for producing a “feature template” from vehicular and EEG signals. This template can be used to generate a feature set utilizing only vehicular signal for evaluating in-vehicle drivers’ MWL.
- Assess the reliability of the feature set developed from the proposed methodology.
- Validate the performance of machine learning (ML) models in quantifying and classifying drivers’ MWL using the features extracted from proposed methodology.

The remaining sections of this article is organized as follows. The background of the research domain and several related works are described in Section 2. Section 3 contains detailed description of the experimental setup, data collection, analysis, feature set generation and validation of the feature set using regression and classification. The outcome of the performed methodologies and discussions on different outcomes are provided in Section 4 and Section 5, respectively. In conclusion, a summary and possible future of this work are discussed in Section 6.

## 2. Background and Related Works

The task of driving is a combination of several dynamic and complex activities that include simultaneous visual, cognitive and spatial tasks [1]. Fastenmeier and Gstalter defined driving as a human–machine system that continuously changes with the environment. The components of the environments are traffic flow (high or low), road layout (straight, junctions, roundabout or curves), road design (motorways, city or rural), weather (rainy, snowy or windy), time of a day (morning, midday or evening), etc. These components define the overall complexity of the driving task [12]. Furthermore, various studies outline driving as a hierarchy of different tasks in three levels. Strategic tasks like decision making constitutes the first level. On the above of strategic tasks, the second level lies with tasks like maneuvering or reacting in response to the change of environment, which is termed the tactical level. The third level is called the operational level, which includes controlling the vehicle. The first two levels demand voluntarily processing and observing various elements of environment by the drivers. On the other hand, tasks on the third level are automatically performed depending on the driver’s experience, which involves less processing of surrounding information. Miscellaneous tasks associated with the primary task i.e., controlling the vehicle, tends to increase the MWL of drivers, which results in errors [1,13].

In the twenty-first century, driving a vehicle causes extensive irregularities in the MWL of drivers [14]. With the increasing number of vehicles on the road and in-vehicle technologies, the task of driving is getting more complex, resulting high MWL. However, the term workload can be related to both physical and/or mental assets and task demands. In case of driving, MWL is more appropriate and considerably varies depending on driver’s capabilities and required task demands [15]. It is observed that both high and low MWL can impede the driving performance [16]. Higher MWL than normal can lead to driver’s diverted attention, distraction, inadequate time and capacity for information processing. On the other hand, low MWL can result slower reaction to events, reduced attention and alertness. Thus, as complex task, driving demands both psychological and physiological undertaking where MWL is an ineluctable aspect [17]. A study dedicated to finding the causes of road accidents demonstrates that human error directly or indirectly contributes to 90% of the accidents [18]. Because of the association of driver’s MWL to committing errors while driving, and since these errors have been demonstrated as a principle contributing factor to road accidents, research on determining the in-drive MWL of drivers looks extremely urgent and important.

### Assessment of Drivers’ Mental Workload

A substantial amount of research works were performed on assessing the MWL of humans while dealing with operational activities, but most of them are concerned about aviation sector rather than automobiles [14]. However, aviation has only a small selection of pilots, which becomes easier to exploit, whereas the automobile domain constitutes with a comparatively higher number of drivers with diverse background, experience, skills and age group, which results in complex research work. Generally, irrespective of the domain, MWL is assessed in different ways. The methods can be assembled into three classes [19].

1. Subjective Measures i.e., NASA Task Load Index (NASA-TLX), workload profile (WP), etc.
2. Task Performance Measures i.e., time to complete a task, reaction time to secondary task, etc.
3. Physiological Measures i.e., EEG, heart rate (HR), etc.

In combination with the subjective measures, the physiological measures are primarily objective in nature, which can be accumulated without imposing additional tasks to the participant. Contrarily, gathering task performance measures requires additional secondary tasks while driving, whereas the primary task remains already overloaded with diverse secondary tasks. Nevertheless, physiological measures can assess the mental impairment of the participant without imposing additional tasks and degrading the performance on primary task [6,8]. According to Guzik, physiological measures are selected often over other measures as a mean of assessing MWL because of cheap and smaller technologies [20]. Respiration, blood pressure, skin conductance, cardiac activities, brain measures, ocular measures, etc., are noteworthy instances of physiological measures. An abundant accessibility of technology, portability and capability of physiological activities, more specifically, indication of the neural activation, EEG signals have been widely chosen by researchers to assess the MWL of drivers while driving. In a recent review of works on drivers’ MWL, Charles and Nixon mention that most research works are carried out using EEG signals as a tool to measure MWL [21,22]. In addition, it has been established through research that a significant association lies between MWL and EEG features extracted in time and frequency domain. Waveform length, zero crossings, mean absolute values, slope signs changes, etc., features are extracted from EEG in a time domain and further utilized in classification tasks in the domain of brain–computer interfacing [23]. On the other hand, the Alpha and the Theta wave rhythms of EEG signals, respectively, over the parietal and the frontal regions of brain significantly illustrate the MWL variation of participants [24,25].

Computationally expensive methods like statistical analysis and signal processing are largely deployed to transform the EEG signals into features that can be directly used for measuring MWL. Literature indicates variety of approaches to extract features from EEG signals. For example, a non-linear approach using fractal dimensions, discrete wavelet transform, non-negative matrix factorization, time and frequency domain analysis, etc. [26,27,28,29,30]. Recently, the use of Deep Learning (DL) techniques increased in this domain to reduce the complexity of adopting the mentioned methods. A Convolutional Neural Network (CNN) was used by Wen et al. for unsupervised feature learning from EEG signals in classifying epilepsy patients [31]. In addition to CNN, use of Long Short-Term Memory (LSTM) [32], Deep Belief Network (DBN) [33], Stacked Denoising Autoencoder (SDAE) [34], etc., were also observed in the literature. After extracting features from the EEG signal, different ML algorithms are widely used, namely, Support Vector Machine (SVM), k-Nearest Neighbors (k-NN), Fuzzy-c Means Clustering, Multi-Layer Perceptron (MLP), etc. [35].

Summarizing, the prevailing methods of assessing in-vehicle MWL of drivers require extensive setup to collect physiological signals. On top of that, complex analysis and computation are required to extract the expected outcome let alone the further deployment of the outcome. However, almost in all modern vehicles, there are provisions available to record the different parameters of vehicle maneuvering e.g., velocity and acceleration. Solovey et al. utilized these vehicular data aligning with physiological data to evaluate automotive user interfaces [9]. As of now, to our knowledge, no work has been done considering only the vehicular data in assessing driver’s MWL. The prior work builds the foundation of this work to employ vehicular data with pre-compiled hybrid template of vehicular and physiological data for assessing in-vehicle MWL of drivers that may be useful to reduce the complexity of in-vehicle setup and extensive analysis of physiological measures.

## 3. Materials and Methods

### 3.1. Experimental Protocol

This study is part of a larger study performed in real driving conditions [24,36,37]. Twenty male participants (24.9 ± 1.8 years old, licensed from 5.9 ± 1 years, with a mean annual mileage of 10,350 km/year) were recruited. They were selected in order to have a homogeneous experimental group in terms of age, sex and driving expertise. The experiment was conducted following the principles outlined in the Declaration of Helsinki of 1975, as revised in 2000 [38]. Informed consent and authorization to use the video graphical material were obtained from each subject on paper, after the explanation of the study.

Two equal cars were used for the experiments, i.e., Fiat 500 L 1.3 Mjt, with a diesel engine and manual transmission. The subjects had to drive the car along a route going through urban roads at the periphery of Bologna (Italy). In particular, the route consisted of three laps of a “circuit” about 2500 m long during the day, with no significant darkness.

According to previous evidence in the scientific literature (Section 2), the difficulty of the driving task was modulated through two variables: road complexity and traffic intensity. In terms of road complexity, the circuit was designed with the aim to include two segments of interest, both about 1000 m long, but different in terms of typology and thus cognitive demand, so named hereafter “Easy” and “Hard”: (i) Easy was a secondary road, mainly straight, with an intersection halfway with the right-of-way, one lane and low traffic capacity, serving a residential area; (ii) Hard was a main road, mainly straight, with two roundabouts halfway, three lanes and high traffic capacity, serving a commercial area. This factor is hereafter named “ROAD”. This assumption was made on the basis of several studies in the scientific literature about road safety and behavior [13,39,40].

In terms of traffic intensity, each participant had to repeat the task two times within the same day, one time during rush and one during normal hours: this factor is hereafter named “HOUR”, while the two conditions are named “Rush” and “Normal”. The rush hours of that specific area have been determined according to the General Plan of Urban Traffic of Bologna (PGTU): the two “Rush hour” time-windows were from 12:30 to 13:30 (lunch time) and from 16:30 to 17:30 (work closing time), with the experiments performed from 9.30 to 17.30, in order to ensure a homogeneous daylight condition. This experimental hypothesis was statistically validated by analyzing, per each subject per each “HOUR”, the number of vehicles encountered along the track as well as the driving speed. The analysis is reported in a previous work obtained from the same experiment [24].

**Figure 1:**
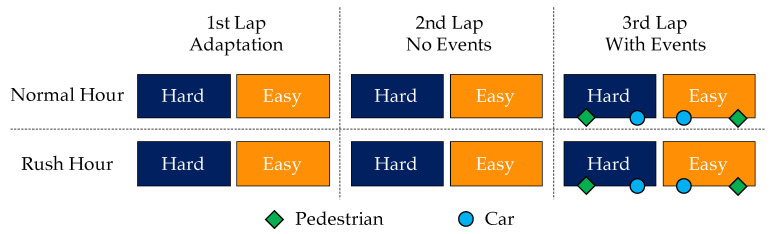
Summary of the experimental protocol. The experiment was carried out with two driving tasks which were different in terms of traffic (Normal and Rush hour) and performed in a randomized order. Each of the driving tasks were comprised of three laps: The 1st lap was intended to make the driver habituated to the circuit and the other (2nd and 3rd) laps were used for analysis. Moreover, events were introduced in the 3rd lap to assess the presence of different scenarios on road when there are absent and present respectively.

To summarize, each subject, after a proper experimental briefing, performed a driving task of three laps along a circuit through urban roads two times, during Rush and Normal hours. Each lap consisted of a Hard and Easy segment, where hard and easy refer to the road complexity, respectively a main and a secondary road, as depicted before. The order of Rush and Normal conditions was randomized among the subjects, in order to avoid any order effect [41]. Also, despite the initial briefing, the first lap of both the tasks was considered an “adaptation lap”, while the data recorded during the second and third laps were taken into account for the analysis. Figure 1 illustrates the overview of the experimental protocol.

During the whole protocol, physiological data, in terms of brain activity through the Electroencephalographic (EEG) technique and eye gazes through Eye-Tracking (ET) devices, and data about driving behavior (vehicular data), through a professional device mounted on the car (i.e., a VBOX Pro), were recorded by guaranteeing time-synchronization among the different devices. In addition, subjective measures of perceived MWL were collected from the subjects after both the tasks through the *NASA Task Load indeX* (NASA-TLX) questionnaire [42]. For the purposes of the present study, only EEG and vehicular data were considered, while eye-tracker and subjective measured were used in previous works to validate experimental design [24,36].

### 3.2. Data Collection

#### 3.2.1. EEG Data Recording and Processing

The EEG signals were recorded using the digital monitoring BEmicro system (EBNeuro, Italy). Fifteen EEG channels (Fpz,Fz,Pz,POz,Oz,AF3,AF4,F3,F4,P3,P4,P5,P6,O1 and O2), placed according to the 10–20 International System, were collected with a sampling frequency of 256 Hz, all referenced to both the earlobes, grounded to the Cz site and with the impedance kept below 20 kΩ. During the experiments, raw EEG data were recorded and the whole processing chain was applied offline. In particular, EEG signal was firstly band-pass filtered with a fourth-order Butterworth infinite impulse response (IIR) filter (high-pass filter cut-off frequency: 1 Hz, low-pass filter cut-off frequency: 30 Hz). The Fpz channel was used to remove eyes-blink contributions from each channel of the EEG signal by using the *Regressive Eye BLINk Correction Algorithm* (REBLINCA) algorithm [43]. This step is necessary because the eyes-blink contribution could affect the frequency bands correlated to the MWL, in particular, the theta EEG band. This method allows us to correct EEG signal, even online [44,45] without losing data and without requiring additional sensors, such as for example electro-oculographic ones.

For other sources of artefacts (e.g., environmental noise and drivers’ movements), specific procedures of the EEGLAB toolbox [46] were employed. Firstly, the EEG signal was segmented into epochs of 2 s (Epoch length), through moving windows shifted of 0.125 s (Shift), thus with an overlap of 0.875 s between two contiguous epochs. This windowing was chosen with the compromise to have both a high number of observations, in comparison with the number of variables, and to respect the condition of stationarity of the EEG signal [47]. In fact, this is a necessary assumption in order to proceed with the spectral analysis of the signal. Then, three automatic methods were applied in order to recognize, and therefore eliminate, artefact epochs [6]: (i) Threshold criterion, recognizing EEG epochs with the signal amplitude exceeding ±100 μV; (ii) trend estimation, once interpolated the EEG epoch, if its slope is higher than 10 μV/s, the considered epoch is marked as “artefact”; (iii) sample-to-sample criterion, recognizing EEG epochs with sample-to-sample differences, in terms of absolute amplitude, higher than 25 μV (i.e., an abrupt variation—nothing physiological happened). The percentage of the rejected data, averaged among the subjects, was 9.3% ± 11% (standard deviation). At the end, the resulting EEG signal was considered “clean”.

From the clean EEG dataset, the Power Spectral Density (PSD) was calculated for each EEG channel for each epoch using the Fast Fourier Transformation (FFT) and a Hanning window of the same length of the considered epoch (2 s length, that means 0.5 Hz of frequency resolution). Then, the EEG frequency bands of interest was defined for each subject by the estimation of the *Individual Alpha Frequency* (IAF) value [48]. In order to have a precise estimation of the alpha peak and, hence of the IAF, a “Closed Eyes” resting condition, one minute long, was recorded for each participant before starting the experimental tasks. Finally, a spectral features matrix (EEG channels × Frequency bins) was obtained in the frequency bands directly correlated to the MWL. In particular, only the theta band [IAF−6÷IAF−2], over the EEG frontal channels, and the alpha band [IAF−2÷IAF+2], over the EEG parietal channels, were considered as variables for the MWL evaluation, as demonstrated in previous scientific literature [49,50,51]. In fact, the ratio between Frontal Theta and Parietal Alpha spectral content is considered as one of the most sensitive biomarkers of human mental workload [51,52,53,54,55]. In particular, in terms of features domain, it consisted of a matrix, for each subject for each epoch, of 187 PSD values (11 EEG channels × 17 bins of frequency − from IAF-6 Hz to IAF + 2 Hz with a resolution of 0.5 Hz −). Actually, only 99 of these features can be selected by the algorithm, because the Regions of Interest are defined a priori: 45 features related to frontal Theta and 54 related to parietal Alpha.

#### 3.2.2. EEG-Based Mental Workload Index Computation

At this point, the automatic-stop-StepWise Linear Discriminant Analysis (asSWLDA), a specific Machine-Learning algorithm (basically an upgraded version of the well-known StepWise Linear Discriminant Analysis) previously developed [6], patented [56] and applied in different applications [25,45,57,58] by some of the authors was employed. On the basis of the calibration dataset, the asSWLDA was able to find the most relevant spectral features to discriminate the MWL of the subjects during the different experimental conditions (i.e., EASY = 0 and HARD = 1). Once it identified such spectral features, the asSWLDA assigns to each feature specific weights $\left(w_{i,train}\right)$, plus a bias $\left(b_{train}\right)$, such that an eventual discriminant function computed on the training dataset $\left(y_{train}\left(t\right)\right)$ would take the value 1 in the hardest condition and 0 in the easiest one. This step represents the calibration, or “Training phase” of the classifier. Later on, the weights and the bias determined during the training phase were used to calculate the Linear Discriminant function $\left(y_{test}\left(t\right)\right)$ over the testing dataset (*Testing phase*), that should be comprised between 0 (if the condition is Easy) and 1 (if the condition is Hard). Finally, a moving average of 8 s (8MA) was applied to the $y_{test}\left(t\right)$ function in order to smooth it out by reducing the variance of the measure: its output was defined as the *EEG-based MWL index* $\left(MWL_{SCORE}\right)$. For the present work, the training data consisted of the Easy segment of the 2nd lap during the “Normal” condition and the “Hard” segment of the 2nd lap during the “Rush” condition ( the two conditions were hypothesized to be characterized by the lowest and highest MWL demand, respectively), while the testing data consisted of the data of the 3rd lap of both the conditions. This hypothesis was validated by previous analysis performed on the same experiment [24].

The training asSWLDA discriminant function (Equation (1), where $f_{i,train}\left(t\right)$ represents the PSD matrix of the training dataset for the data window of the time sample *t*, and of the *i*th feature), the testing one (Equation (2), where $f_{i,test}\left(t\right)$ is as $f_{i,train}\left(t\right)$ but related to the testing dataset) and the equation of the *EEG-based MWL index* computed with a time-resolution of 8 s ($MWL_{SCORE}$, Equation (3)), are reported.

(1) $$\begin{array}{ccc} y_{train}\left(t\right) & = & \sum_{i}w_{i,train}\cdot f_{i,train}\left(t\right)+b_{train} \end{array}$$

(2) $$\begin{array}{ccc} y_{test}\left(t\right) & = & \sum_{i}w_{i,train}\cdot f_{i,test}\left(t\right)+b_{train} \end{array}$$

(3) $$\begin{array}{ccc} MWL_{SCORE} & = & 8MA(y_{test}\left(t\right)) \end{array}$$

#### 3.2.3. Vehicular Data

Each car was equipped with a Video VBOX Pro (Racelogic Ltd, Buckingham, UK), a system able to continuously monitor the cinematic parameters of the car, integrated with GPS data and videos coming from up to four high-resolution cameras. The system was fixed within the car, at the center of the floor of the back seats, in order to put it as close as possible to the car barycenter, while two cameras were fixed over the top of the car. The system recorded car parameters (i.e., velocity, acceleration, lateral and longitudinal acceleration) with a sampling rate of 10 Hz.

**Figure 2:**
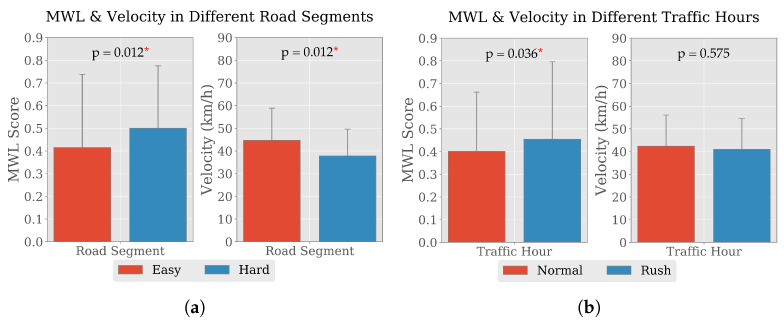
Average Mental Workload (MWL) score and velocity of nine participating drivers in different (**a**) Road segments and (**b**) traffic hours. The standard deviations are indicated, the *p*-values obtained from the two-sided Wilcoxon signed-rank tests are presented and significant values at 5% confidence interval are marked with asterisks (*).

With the availability of the vehicular data, its nature was investigated at the group level with respect to different traffic situations, road conditions, presence of events and type of events. Moreover, the change in MWL of drivers were also studied alongside and prominent trend of changes were observed. In the exploratory analysis, comparison of mean values and two-sided Wilcoxon signed-rank tests [59] were performed considering the null hypothesis, $H_{0}$: “there is no difference between the observations of the two measurements” and the alternate hypothesis, $H_{1}$: “the observations of the two measurements are not equal” with level of significance of 0.05. Figure 2 illustrates the change in drivers’ average MWL score and velocity in different traffic hour and road conditions along with the standard deviations. A two-sided Wilcoxon signed-rank test was used to analyze the MWL of drivers on Easy and Hard segments of the track to test if the change in segment had a significant effect on the MWL. Drivers’ MWL while driving on the Easy segment was lower (0.42±0.32) compared to the Hard segment (0.51±0.27); there was a statistically significant increase in blood pressure (t=0.0,p=0.012). Conversely, on the Easy segment of the track, participating drivers maintained average velocity 44.69±14.21 kilometers per hour (km/h) whereas the average velocity dropped to 37.81 ± 11.83 km/h on the Hard segment. A two-sided Wilcoxon signed-rank test on the driving velocities of all the participants for the Easy and Hard segments produced t=0.0,p=0.12, which signifies the difference of velocity due to different road segments. A similar trend of increasing MWL was observed while drivers drove during Normal (0.40±0.26) and Rush (0.45±0.34) hours. A two-sided Wilcoxon signed-rank test on drivers’ MWL for driving during different hours produced t=3.0,p=0.036, signifying the change in MWL. On the other hand, average driving velocity during Normal hour was 42.39 ± 13.70 km/h, which reduced to 40.98 ± 13.57 km/h in Rush hour. According to the result of a two-sided Wilcoxon signed-rank test (t=14.0,p=0.575), there were no significant difference between driving velocity during Normal and Rush hour.

**Figure 3:**
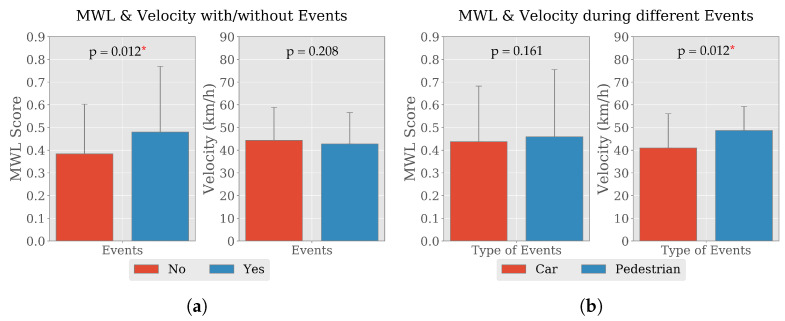
Average MWL score and velocity with standard deviation calculated from the data of nine participating drivers with respect to events. Sub-figure (**a**) illustrates the variation of MWL score and velocity with/without the presence of events and Sub-figure (**b**) illustrates the the effect of car and pedestrian on MWL score and velocity. The *p*-values obtained from the two-sided Wilcoxon signed-rank tests are presented and significant values at 5% confidence interval are marked with asterisks (*).

Two different events; a car and a pedestrian, were introduced during the 3rd lap driving with a view to mimic the general road users and observe their effect on drivers’ MWL and vehicle handling. Comparative investigation, thus considering the third lap with respect to the second one (without any event) revealed that MWL of drivers increase about 30%. Drivers’ average MWL with no additional event was 0.38±0.22 whereas average MWL increased to 0.48±0.29 in presence of simultaneously participating road users. A two-sided Wilcoxon signed-rank test indicated that drivers’ MWL with no event were statistically significantly lower than the MWL while driving with events t=0.0,p=0.012. On the other hand, the average driving velocity without events was 44.33 ± 14.52 km/h and in presence of events the average velocity was 42.77 ± 13.80 km/h which came out as statistically insignificant (t=9.0,p=0.208). The difference in type of events has not affected the average MWL of drivers, 0.44±0.25 in presence of car and 0.46±0.30 in presence of pedestrian. From the outcome of the Wilcoxon test (t=8.0,p=0.161), the change is also statistically insignificant. Again, the average velocity was lower in presence of the a car 40.98 ± 15.06 km/h than a pedestrian 48.67 ± 10.53 km/h. A two-sided Wilcoxon signed-rank test indicated that the change in average velocity was statistically significant (t=0.0,p=0.012). Illustrations of the change in average MWL and driving velocity with standard deviation due to presence and type of events are presented in Figure 3.

The aforementioned group-level analysis on drivers’ MWL and driving velocity demonstrated significant change in MWL due to change in the driving environment. For the driving velocity, the two-sided Wilcoxon signed-rank tests did not produce satisfactory *p* values to signify the differences except the case with different types of events. However, the performed analysis partially contrives the need to formalize the relationship between MWL and vehicular data. In a way to further formalization, physiological data were used collectively with vehicular data to accumulate more objective knowledge since vehicular data does not represent direct measures for MWL estimation of drivers.

### 3.3. Mutual Information Based Feature Extraction

To support the primary assumption on assessing MWL using mostly vehicular data, the first part of the analyses contains extraction of MI [11] between EEG and vehicular data. In the process of extracting MI, entropy and conditional entropy of the variables were calculated using Corollary 1 to Theorem 1 [11], which are subsequently presented below.

**Theorem** **1.** *Given continuous random variable $X\in R^{d}$ representing available variables or observations and a continuous valued random variable Y representing class labels. The uncertainty or entropy in drawing one sample of Y at random according to Shannon’s definition:*

(4) $$H\left(Y\right)=E_{x}\;[log_{2}\frac{1}{p(y)}]\;=-\int_{x}p\left(x\right)\;log_{2}\left(p\left(x\right)\right)\;dx$$

*After having made an observation of a variable vector x, the uncertainty of the class identity is defined in terms of the conditional density p(y|x):*

(5) $$H\left(Y|X\right)=\int_{x}p\left(x\right)\left(-\int_{y}p\left(y|x\right)\;log_{2}\left(p\left(y|x\right)\right)\;dy\right)dx$$

*Reduction in class uncertainty after having observed the variable vector x is called the mutual information between X and Y, same as the Kullback–Leibler divergence between the joint density p(y,x) and its factored form p(y)p(x).*

(6) $$\begin{array}{ccc} I(X,Y) & = & H(Y)-H(Y|X) \end{array}$$

(7) $$\begin{array}{cc} \;= & \int_{y}\int_{x}p\left(y,x\right)\;log_{2}\frac{p(y,x)}{p(y)\;p(x)}\;dx\;dy \end{array}$$

We derived a template for producing feature set using solely vehicular signal using Corollary 1 which was derived from Theorem 1.

**Corollary** **1.** *Given a continuous random variable E representing EEG observations and a continuous random variable V representing vehicular signals, from a specific population distribution and representing the objective and indirect measure of MWL, respectively. The mutual information I(E,V) between variables E and V represents the mutual dependency between them by quantifying the amount of information they share collectively for estimating MWL, which can be derived using corresponding variable vectors e and v.*

**Figure 4:**
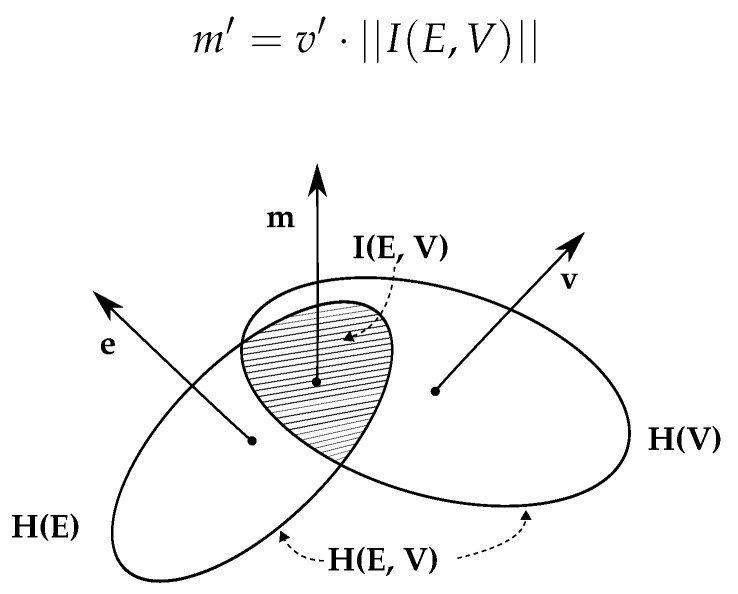
Illustration of shared information between Electroencephalography (EEG) and vehicular signal spaces.

In association with the Corollary 1, for better visualization, Figure 4 illustrates the concept of MI with respect to the variables used in this study. *E* for EEG and *V* for vehicular data are depicting *X* and *Y* as described in Theorem 1. Entropy value for vehicular data and EEG are represented with H(V) and H(E). Joint entropy H(E,V) consists of the union of the entropy spaces and mutual information I(E,V) in the intersecting space. Thus, H(E,V)=H(E)+H(V)−I(E,V) is derived using Set Theory. *e* and *v* represent a single instance of EEG and vehicular signal, respectively, and *m* represents a single instance of I(E,V), which is the mutual information shared by single instances *e* and *v*. Formally, I(E,V) is a matrix of order p×q, where *p* and *q* are the number of vehicular and EEG features. respectively. Each row of the matrix represents the shared information between a single vehicular feature and every EEG features. Furthermore, ||I(E,V)||, the norm of each row of I(E,V) was calculated which is a vector containing the collective magnitude of the shared information between each vehicular feature and all EEG features. The ||I(E,V)|| was further used to calculate new MI-based feature vector $m^{\prime }$ from vehicular features entirely with the following equation where $v^{\prime }$ is a new instance vector of vehicular features.

(8) $$m^{\prime }=v^{\prime }\cdot \left||I\left(E,V\right)|\right|$$

In the extraction of MI-based features from the data of this particular study, data were represented in vector forms i.e., *e* for EEG and *v* vehicular data, which belongs to the domains *E* and *V*, respectively. Formally, $E,V\in R^{d}$, where *d* is bears 45 and 4, respectively, for this study. For this specific analysis, the EEG signal was analyzed again. In fact, in the previous section of the study we employed a well-established approach, even patented [56], to obtain the EEG-based MWL reference measurements [6,24,50]. In that case, a specific a priori hypothesis (only frontal Theta and parietal Alpha features) and processing procedures (e.g., automatic artifacts correction/removal) were necessary for the classification algorithm reliability and the possibility of employing it even online [44]. In this second analysis, because of the absence of these restrictions, we preferred to employ more complex artifacts rejection algorithms and to enlarge features domain all the EEG channels throughout the scalp were considered while extracting the features. At first the raw EEG data were cleaned i.e., the artefacts were removed using ARTE (Automated aRTifacts handling in EEG) [60] and subsequently, 45 features were extracted from power spectral density values. The IAF value was determined as the peak of the general alpha rhythm frequency (8–12 Hz). Subsequently, the average frequency of the theta band [IAF−6,IAF−2], the alpha band [IAF−2,IAF+2] and the beta band [IAF+2,IAF+18], over all the EEG channels were calculated. Table 1 shows the mapping between the features are frequency rhythms. On the other hand, the vehicular signal was resampled to the sampling frequency of the EEG signals in order to synchronize and generate equal number of data points to analyze. The steps of the process are as follows: the vehicular signal at 10 Hz was at first upsampled by 256. After that, a zero-phase low-pass finite impulse response (FIR) filter was applied and then the signal was downsampled by 10. As a result, the resulting sample rate became 256 Hz i.e., 256/10 times the original sample rate 10 Hz. The vehicular feature set contains the values for velocity, acceleration, lateral and longitudinal acceleration signals. Finally, values of all the features gathered from vehicular and EEG signals were normalized with the min–max feature scaling within the range 0 to 1, in order to restrict the ML algorithms to pick up unimportant characteristics from the data due to difference in values of different features.

**Figure 5:**
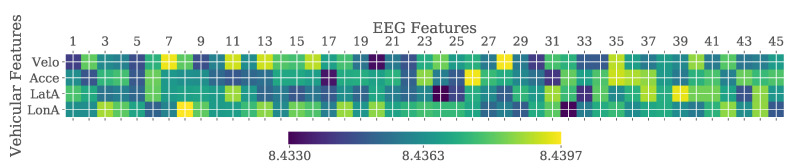
Calculated MI values between EEG and vehicular signal. The columns of the matrix correspond to 45 features extracted from EEG signals and the rows corresponds to four vehicular features: Velocity (Velo), Acceleration (Acce), Lateral Acceleration (LatA) and Longitudinal Acceleration (LonA). The color bar below illustrates the range of values for each pair of EEG and vehicular features where dark blue on the left corresponds to low mutual information and gradually higher mutual information values towards right are represented by yellow.

Considering all of the available vehicular features and the calculated features from EEG signal, MI values were calculated using Equation (7). The associated MI values, illustrated in Figure 5, demonstrates the shared knowledge between vehicular data and EEG data. Though the range of the MI values are not significant, yet they share some information, which leverages the motivation to use MI values in further classification or quantification of MWL in this work. Finally, an MI-based feature set was constructed using Equation (8). Table 2 represents the number of features from different feature sets which were considered in further stages of this study. Here, the prime concern of the study is to investigate the performance of MI-based features in MWL assessment and EEG features are used as an established objective measure reference.

### 3.4. Prediction and Classification Models

To evaluate the MWL of drivers from features developed with MI between EEG and vehicular features and compare its performance while assessed with solely EEG-based feature set, ML algorithms of a different nature from a functional point-of-view were trained. During the prediction task, expert-defined MWL scores (Section 3.2.2) were used as true predictions to train the regression models. On the other hand, for the classification tasks, two sets of binary classes were considered. In terms of MWL classification, data instances were labeled as *High* and *Low* following the factors, “ROAD” and “HOUR” described in Section 3.1. To examine the use of the extracted MI-based features in classification tasks other than MWL, another binary classification task was performed assuming the two events; *Car* and *Pedestrian*, which were introduced in Lap 3 during the experiment as true labels. The ML algorithms, which were used in different prediction and classification tasks, are described briefly below.

Regression is the simplest supervised ML model that estimates the relationship between an independent and a dependent variable with statistical analyses [61]. Generally, Linear Regression (LnR) and Logistic Regression (LgR) are deployed for predicting continuous and binary categorical values, respectively, which aligns perfectly with this study. For both regression and classification tasks, normalized data were used. Moreover, for classification, LgR was performed with balanced class weights and L2 regularization.

Multilayer Perceptron (MLP) [62] is a subclass of Artificial Neural Network (ANN) with at least three layers of nodes—an input layer, hidden layer and output layer. Here, MLPs were trained for both classification and regression tasks with three hidden layers of 32, 16 and 4 nodes, respectively, Rectified Linear Unit (ReLU) activation, Adam optimizer and batch size 128.

Random Forest (RF) is an ensemble method, which builds a collection of randomized decision-trees developed from bootstrapped data points and predicts on the basis of majority voting from all the trees for classification tasks [63] whereas for regression tasks, it takes the average of prediction. In addition to that, RF operates with an underlying feature selection method which removes non-important features for prediction tasks automatically. RF was implemented using bootstrapping as the ensemble method.

The working principle of Support Vector Machine (SVM) concentrates mostly on finding the hyper-plane, which simultaneously minimizes the empirical classification error and maximizes the geometric margins in the classification tasks [64]. SVM transforms the true data points from the input space to high dimensional space that facilitates the classification task by determining a decision boundary. For prediction or regression tasks, the decision boundary is used to predict the continuous value or target value. SVM-based regression and classification models have a very good generalization capability on multidimensional data and dynamic classification/prediction scheme, which makes them appropriate for the concerned tasks. Moreover, literature shows deliberate use of SVM in the domain of EEG signal analysis and MWL assessment [35,65,66]. In this study, for all tasks, the SVM was configured with Radial Basis Function (RBF) kernel with degree 3. By trial and error, the final regularization parameter C was set to 1.0 and epsilon to 0.2 as the model parameters.

The trained ML models were further deployed in performing different tasks to evaluate the MI-based features. The model parameters used to train different models for respective tasks are summarized in Table 3.

The evaluation of the features extracted through proposed approach was conducted in several steps: (1) predicting MWL score, (2) classifying MWL and (3) classifying events. For the prediction task, the evaluation was performed using 10-fold Cross Validation (CV) and Leave-One-Out (LOO) (subject) validation. In the process of 10-fold CV, the whole dataset was divided into 10 equal sets. After that, 10 iterations of training and testing of aforementioned ML models were performed considering each of the divided sets as test set and rest nine sets as training set. So, the ratio between training and validation was 90% and 10%. The repetition of the experiment was conducted 10 times and average results are presented in the manuscript. On the other hand, in LOO-subject validation, there were 9 (number of subjects) iterations. In each iteration, the training set consisted of 8 participants and the left-out subject’s data was taken as a test set. In both of the validation approach the average split of training and testing ratio was approximately 90:10. In the case of the classification task, 10% of all data points were selected through stratified sampling as a holdout test set. The rest of the data were further used for training and validating the models using the two described validation methods with a view to flag problems like overfitting or selection bias.

The tasks of implementation of the proposed methodology and representation of result were done using Python [67] and R [68] environments. Python libraries NumPy [69] and Pandas [70] were invoked for preparing the data. ML models were trained, validated and tested using the Scikit Learn [71] library for Python. The plots and graphs were drawn utilizing different methods of Matplotlib [72]. Statistical tests were conducted mostly using methods from SciPy [73] library for Python and pROC [74] package for R.

## 4. Results

The outcome of the performed study is presented from the viewpoint of two different tasks: prediction and classification. In the process, the developed prediction models were evaluated using Mean Absolute Error (MAE) and Mean Standard Error (MSE). The evaluation of the developed MWL and event classifiers were done in terms of confusion matrices, Receiver Operating Characteristic (ROC) curves, accuracy, sensitivity and specificity. In addition to the mentioned performance measures, balanced accuracy was also measured since both of the classification task of this study were binary classification and due to division of epochs from the signal recordings and duration of driving, the number of instances representing each class varied to some extent.

### 4.1. Quantification of Drivers’ Mental Workload

**Figure 6:**
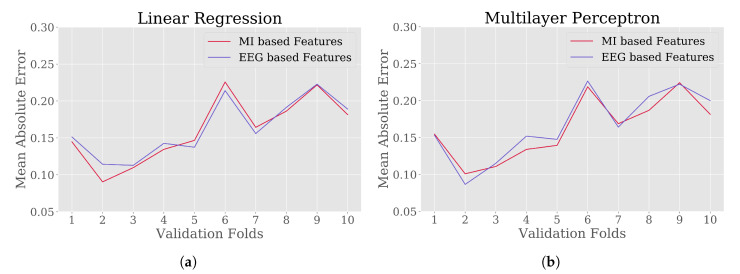
The 10-fold Cross Validation (CV) score in terms of Mean Absolute Error (MAE) for regression models: (**a**) Linear Regression (LnR) and (**b**) Multilayer Perceptron (MLP), where the expert derived MWL scores were considered as true values. For each of the models, two different sets of features were used (Table 2).

**Figure 7:**
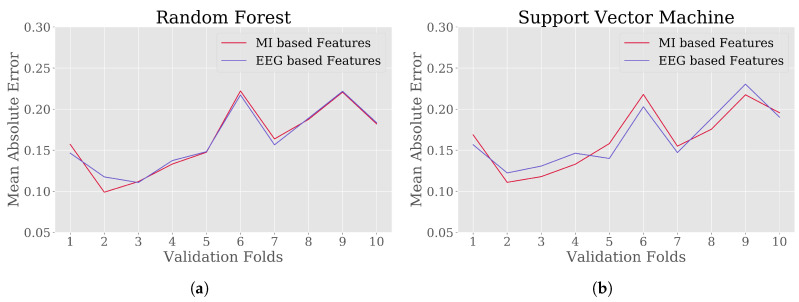
The 10-fold CV score in terms of MAE for regression models: (**a**) Random Forest (RF) and (**b**) Support Vector Machine (SVM), where the expert derived MWL scores were considered as true values. For each of the models, two different sets of features were used (Table 2).

Four different prediction models LnR, MLP, RF and SVM were trained with expert defined MWL scores against EEG and MI based features. The performance of the models were validated with 10-fold CV approach. Figure 6 and Figure 7 illustrate the MAE values for 10 folds of validation sets in predicting MWL scores from EEG and MI based feature set. An overview of the prediction scores of each model on two different feature sets are provided in Table 4 from the performed CV.

### 4.2. Drivers’ Mental Workload and Event Classification

Primarily, the MI-based feature set was tested in MWL classification against the EEG-based feature set with the respective models described in Table 3. For MWL classification, the *Low* MWL was considered a positive class and *High* MWL was considered a negative class. In addition to MWL classification, the event classification tasks were performed to establish the use of MI-based features in other classification tasks, which was inspired from the result obtained in MWL classification. In event classification, *Car* and *Pedestrian* events were defined as the positive and negative classes, respectively, to measure the performance. For both the classification tasks, 10-fold CV and LOO-subject CV were used to train the models on different feature sets. The models used in MWL classification were used to train with the labels of events keeping the model parameters unchanged with a view to conduct comparative assessment.

In order to evaluate the classification performance of the aforementioned classifiers, a one-sided Wilcoxon signed-rank test [59] was performed. For a single classifier, the two sets of performance measures of classification where trained with MI-based features and with EEG-based features were considered and the test was conducted. The null hypothesis, $H_{0}$: There is no difference in average performance measures of a classifier when trained with MI-based and EEG-based features; the alternate hypothesis, $H_{1}$: The average performance measures of the classifier trained with MI-based features are higher than trained with EEG-based features. The test hypotheses are mathematically outlines in the expressions below.

(9) $$\begin{array}{cc} H_{0}: & \mu_{MI}=\mu_{EEG} \end{array}$$

(10) $$\begin{array}{cc} H_{1}: & \mu_{MI}>\mu_{EEG} \end{array}$$

The result is summarized in Table 5, where it can be observed that, while classifying MWL, only SVM achieved significantly higher performance while trained with the MI-based features. On the other hand, all the classifiers performed better while trained with MI-based features than EEG-based features in classifying events.

**Figure 8:**
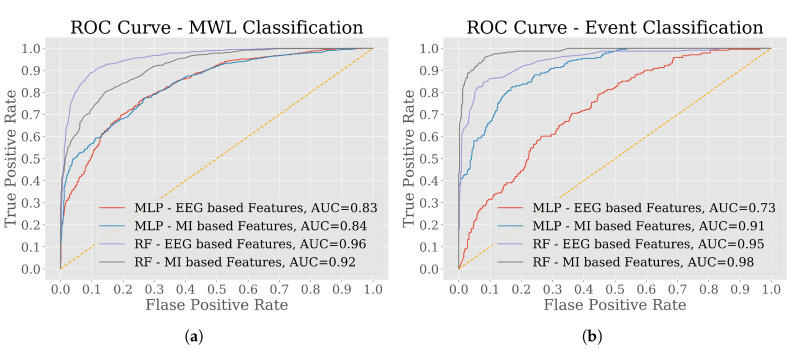
Receiver Operating Characteristic (ROC) curves for the best two classifier models among Logistic Regression (LgR), MLP, SVM and RF. The classifiers were deployed in two different binary classification tasks: (**a**) *Low* or *High* MWL and (**b**) type of events—*Car* or *Pedestrian*. For each of the tasks, all the classifier models were trained using 10-fold cross-validation approach.

ROC curves associated with Area Under the Curve (AUC) values, for both of the classification tasks are illustrated in Figure 8. The ROC curves were drawn for the holdout test set. In both of the tasks, from the overall perspective, RF classifier outperformed other classifiers with both feature sets in terms of AUC values. Specifically, in MWL classification, the accuracy was higher for using EEG-based feature set but in event classification MI-based feature set produced higher AUC value.

In addition to the calculated AUC values from different performance metrics, 95% Confidence Interval (CI) of true AUC, *Z* and *p* values were extracted from Delong’s test of comparing AUC values. To conduct the test, the null hypothesis was set as, $H_{0}$: and alternative hypothesis, $H_{1}$: “the values of AUC for classifiers trained on MI-based features are higher than the values of AUC for classifiers trained on EEG-based features”. Table 6 presents the results of DeLong’s test, which is similar to the results obtained from one-sided Wilcoxon signed-rank test outlined in Table 5 in terms of rejecting the null hypothesis $H_{0}$ with significance level 0.05.

**Figure 9:**
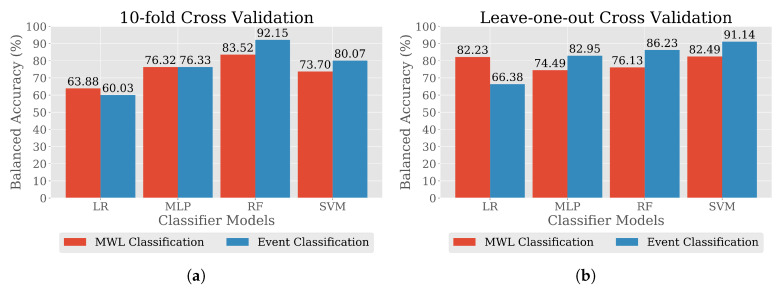
Maximum balanced accuracy in different CV method for MWL and event classification using MI-based features by different classifier models: (**a**) 10-fold CV and (**b**) Leave-One-Out (LOO)-subject CV.

The test classification report for MWL classification is presented in Table 7. In addition to that, Table 8 provides the classification report on the holdout test set, which demonstrates improvements in performance accuracy for classification using MI-based features. To assess the solitary performance of classifiers trained with MI-based features, the maximum accuracy achieved in different CV approach over all the data splits were investigated. Figure 9 illustrates bar charts developed with the maximum accuracy achieved by different classifiers in classifying MWL and events with MI-based features. It can be observed that, in 10-fold CV, the highest accuracy was 92.15% from RF classifier, whereas in event classification, SVM achieved 91.14%, which is the highest of all other classifiers while considering LOO-subject CV.

## 5. Discussion

An increase of secondary tasks e.g., reaching for the mobile phone, interacting with the mobile phone (touching on the screen, dialing and texting), talking, reading the screen, glancing at the phone momentarily and talking or listening to a hands-free device together with the primary task of driving causes increased MWL. According to the state-of-the-art (SotA) approaches, to measure MWL, Electroencephalography (EEG) has been proven to be a good parameter and widely used in research [6,7,8], although it is not feasible enough in terms of data acquiring, processing and decision making while driving a car in naturalistic environment. So, the aim of this study is to perform research and development to identify a methodology for constructing a novel mutual information-based feature set from the fusion of electroencephalography and vehicular signals and deployed in evaluating drivers’ mental workloads. In this study, EEG and vehicular signals were recorded through driving experiment in real scenarios that varies in different factors; “HOUR” and “ROAD” [24]. Here, two different events were also introduced to investigate the effects on drivers’ MWL. Since the experiment was conducted in a real environment, there might be the presence/absence of other road users. The events leveraged the provision for analyzing uniformly for all participants the effect of specific road users other than the regular traffic on the road. According to the initial data analysis at group level, it was observed that different situations and road users affect the MWL of drivers and their vehicle handling. The results results from the observation (Section 3.2) confirmed the experimental hypothesis, i.e., “the driving task in terms of road complexity as well as events induced differences in driving behaviors and drivers’ experienced MWL”. Statistical hypothesis tests were conducted on average driving velocity and drivers’ MWL and significant (*p* < 0.05) differences were observed. The tests are described in details in Section 3.2.3. In addition to that, several comparative plots were drawn to assess the effects visually, which are illustrated in Figure 2 and Figure 3. In short, the comparisons pointed out that MWL and vehicle handling both changes when the road condition or events on the road are altered. However, the effects of change in events on MWL and driving behaviors are stronger than change in road condition. These findings and together with prior literature review on use of advantages and disadvantages of EEG features as a measure of MWL produced the base of further analysis and increase the urge to utilize mostly vehicular features in association to EEG for evaluating MWL of drivers.

To combine EEG features and vehicular features, a correlation between them were calculated and the assessed values of the correlation coefficients were negligible. On the contrary, prior investigations on the average driving velocity and MWL (Section 3.2) showed changes while driving environments were varied (Section 3.2.3). Thus, the motivation of exploiting MI between EEG and vehicular signal developed entirely on the low correlation coefficient and conversely significant similarity in the change of MWL and vehicular signal. Furthermore, the new novel concept of utilizing MI was proposed. Here, the reference values of MI between two continuous variables should be in the range [1,∞] [11]. The MI is calculated based on the relation between EEG and vehicular features where the average value was found to be approximately 8.5, which is very low but not null. The data for this study were recorded from a specific experiment from some specific participants, which represented their brain activity and vehicle handling together for the respective population distribution. However, The low MI values could be derived due to a smaller number of vehicular features. Despite the fact that the MI values were low, in MWL evaluation, the proposed features in some cases outperformed established objective measures. If there were more vehicular features, there could be wider variety of ways to mimic the handling of vehicle by the participants. As a result, systems would attain higher performance in MWL evaluation. Experiments are underway to increase the number of vehicular features by adding other parameters from inertial measurement unit (IMU) devices.

One of the objectives of this study was to quantify MWL of drivers from the proposed feature set. To test the performance of using the proposed feature set, four different ML regression methods were investigated: LnR, MLP, RF and SVM, considering the MWL score extracted by expert-defined methods as true values. For the regression, the true values of MWL score fall in the range [0,1], where 0 represents no MWL and 1 represents highest from individual point of view [24]. For each of the regression models, the average MAE and MSE were around 0.16 and 0.04 (Table 4). Again, these errors were compared with the results of regression models trained using EEG-based features. In comparison, using different features produced approximately similar errors while predicting MWL scores of drivers and the comparison of MAE in 10-fold CV is illustrated in Figure 6 and Figure 7. From the visualizations it was observed that the difference in average error from RF regression model was lowest among the considered models, which might be an effect of functional differences in terms of ensemble technique [63], as described in Section 3.4.

In addition to MWL quantification, the performances of MWL and event classification using MI-based features were also examined against EEG-based features. Classifier-wise average performance on MWL and event classification was tested using a one-sided Wilcoxon signed-rank test [59]. Unlike MWL quantification, the average performance of SVM classifier with MI-based feature set was significantly higher in both classification tasks (Table 5). According to Shah, SVM is the most widely-used algorithm for classification tasks on the basis of features extracted from EEG signals [35]. The initial finding of this study aligns with the statement. On the other hand, the other three classifiers: LgR, MLP and RF performed better in event classification with MI-based features. To access the correct binary classification capacity, AUC-ROC curves were plotted where RF outperformed all other classifiers in terms of AUC values. Figure 8 illustrates the AUC-ROC curves for RF and MLP classifiers that achieved the higher AUC values while tested on the holdout set for simplicity. In addition to that, DeLong’s test [75] of comparing AUC values demonstrated similar significant differences as the one-sided Wilcoxon signed-rank test [59] showed. It can be observed from Table 6 that all the calculated AUC values are within the 95% confidence interval for true AUC values. Moreover, the values of *Z* and *p* are consistent i.e., in case of significant values of *p*, we accept the alternate hypothesis that the values of AUC for classifiers trained on MI-based features are higher than the values of AUC for classifiers trained on EEG-based feature and the signs of test statistics, *Z* express the same relation between the AUC values. However, according to the performance metrics, in MWL classification, RF achieved the highest AUC value of 0.92 with accuracy 82% with MI-based features and the AUC value was 0.96 (Figure 8a) with accuracy 88% (Table 7) with EEG-based features. Again, the performance on event classification (*Car* or *Pedestrian*) was evaluated with the same ML algorithms considering both the feature sets. In event classification result, RF with MI-based features with AUC value 0.98 outperformed EEG-based features with AUC value 0.95 (Figure 8b). The accuracy on the test set in the classifying event was found to be 94% by the RF classifier by using MI-based features, which is the best performance achieved in this whole study (Table 8).

## 6. Conclusions

In conclusion, the present study was carried out through a driving experiment in a real environment, which was aimed at investigating the utilization of vehicular signals in evaluation of MWL of drivers with a view to reduce the effort of using EEG signals and eliminate the task of managing redundant EEG signal recording apparatuses. This paper presents an MI-based feature set construction methodology with the combination of EEG and vehicular signals. The feature set was deployed to evaluate drivers’ MWL in terms of score and labels. Several ML models were trained to perform the evaluation tasks. The values of MAE in MWL score prediction showed that there was approximately no difference between the predicted score generated using MI-based features and EEG features. On the other hand, in classification tasks, it was observed that RF classifiers performed better than other classifiers in labeling MWL and events in terms of performance metrics of ML models, but through statistical tests it was observed that SVM performed significantly better than all other classifiers. While classifying MWL, the highest accuracy observed was 88% with EEG-based features and 82% with MI-based features. Furthermore, using MI-based features outperformed EEG-based features in two specific events (a pedestrian crossing the road and a car entering in the traffic flow) classification with an accuracy of 94%. Though the accuracy in MWL classification from the developed feature set was not equivalent to EEG features, the accuracy in event classification urges the need of re-evaluation of the proposed fusion methodology of feature extraction with higher number of vehicular features in future studies.

## Figures and Tables

**Table 1:** Mapping among different EEG channels, three significant frequency rhythms and identifications (ID) of features. Each row represents the IDs of the features extracted from specific frequency rhythm from the EEG channels mentioned in the table head.

Rythms	FPz	Fz	Pz	POz	Oz	AF3	AF4	F3	F4	P3	P4	P5	P6	O1	O2
**theta** (θ)	1	4	7	10	13	16	19	22	25	28	31	34	37	40	43
**alpha** (α)	2	5	8	11	14	17	20	23	26	29	32	35	38	41	44
**beta** (β)	3	6	9	12	15	18	21	24	27	30	33	36	39	42	45

**Table 2:** List of different feature sets and corresponding number of features used for validating the proposed methodology.

Feature Set	Number of Features
EEG-based	45
MI-based	4

**Table 3:** Parameters used in building different models for prediction and classification tasks.

ML Models	Parameter Details	Task
Linear Regression	Intercept fit: True	Prediction
(LnR)	Normalize: True	
Logistic Regression	Intercept fit: True	Classification
(LgR)	Normalize: True	
	Class weight: Balanced	
	Regularization: L2	
Multilayer Perceptron	Hidden layers: 32, 16, 4	Prediction & Classification
(MLP)	Activation: ReLU	
	Optimizer: Adam	
	Batch size: 128	
Random Forest	Estimators: 100	Prediction & Classification
(RF)	Bootstap: True	
	Maximum depth: 5	
Support Vector Machine	Kernel: RBF	Prediction & Classification
(SVM)	Degree: 3	
	C: 1.0	
	Epsilon: 0.2	

**Table 4:** The 10-fold CV summary in terms of Mean Absolute Error and Mean Squared Error for predicting MWL score using EEG and Mutual Information (MI)-based features.

Model	Features	MAE			MSE		
		Minimum	Maximum	Average	Minimum	Maximum	Average
LnR	EEG-based	0.11	0.22	0.16	0.02	0.07	0.04
	MI-based	0.09	0.23	0.16	0.02	0.07	0.04
MLP	EEG-based	0.09	0.22	0.17	0.02	0.07	0.04
	MI-based	0.10	0.22	0.16	0.02	0.06	0.04
RF	EEG-based	0.11	0.22	0.16	0.02	0.07	0.04
	MI-based	0.10	0.22	0.16	0.02	0.07	0.04
SVM	EEG-based	0.12	0.23	0.17	0.03	0.07	0.04
	MI-based	0.11	0.21	0.17	0.02	0.06	0.04

**Table 5:** Summary of one-sided Wilcoxon signed-rank tests [59] on the average performance in 10-fold CV of classification tasks by different classifiers trained with MI and EEG based features. The significant values i.e., p<0.05, are marked with asterisks (*).

Tasks	Classifiers							
	LgR		MLP		RF		SVM	
	t	p	t	p	t	p	t	p
**MWL Classification**	0.0	0.994	9.0	0.896	0.0	0.994	36.0	0.006 *
**Event Classification**	32.0	0.025 *	36.0	0.006 *	33.0	0.018 *	36.0	0.006 *

**Table 6:** Summary of DeLong’s test [75] to compare Area Under the Curve (AUC) values at significance level 0.05 (5.00 × 10^−2^). The values were summarized for LgR, MLP, RF and SVM classifiers in different classification tasks on the holdout test set. The significant values i.e., p<0.05, are marked with (*).

Model	Features	MWL Classification				Event Classification			
		AUC	95% CI	*Z*	*p*	AUC	95% CI	*Z*	*p*
LnR	MI-based	0.70	0.68–0.73	−1.727	9.58 × 10^−1^	0.76	0.73–0.80	4.005	3.10 × 10^−5^ *
	EEG-based	0.73	0.71–0.75			0.65	0.61–0.69		
MLP	MI-based	0.86	0.84–0.87	−0.212	5.84 × 10^−1^	0.90	0.88–0.92	7.606	1.42 × 10^−14^ *
	EEG-based	0.86	0.84–0.88			0.73	0.69–0.77		
RF	MI-based	0.92	0.90–0.93	−5.540	1.00 × 10^0^	0.98	0.98–0.99	4.060	2.44 × 10^−5^ *
	EEG-based	0.96	0.95–0.97			0.94	0.93–0.96		
SVM	MI-based	0.82	0.80–0.84	8.715	2.2 × 10^−16^ *	0.87	0.85–0.90	3.096	9.84 × 10^−4^ *
	EEG-based	0.69	0.67–0.72			0.80	0.77–0.84		

**Table 7:** Performance summary of classifying *Low* and *High* MWL with LgR, MLP, SVM and RF classifier models using EEG and MI-based feature on the holdout test set. In this task, the total number of observations was 1710, where low MWL was considered as the positive class. The number of observations with positive and negative class were 917 and 793, respectively. The highest accuracies obtained by using different feature sets are marked with (*).

Criteria	Using EEG-Based Features				Using MI-Based Features			
	LgR	MLP	SVM	RF	LgR	MLP	SVM	RF
**True Positive**	736	776	342	864	688	715	576	783
**False Negative**	181	141	575	53	229	202	341	134
**False Positive**	362	293	138	157	410	230	146	175
**True Negative**	431	500	655	636	383	563	647	618
**Sensitivity**	0.80	0.85	0.37	0.94	0.75	0.78	0.63	0.85
**Specificity**	0.54	0.63	0.83	0.80	0.48	0.71	0.81	0.78
**Precision**	0.67	0.73	0.71	0.85	0.63	0.76	0.80	0.82
**Recall**	0.80	0.85	0.37	0.94	0.75	0.78	0.63	0.85
$F_{1}$ **score**	0.73	0.78	0.50	0.89	0.68	0.77	0.70	0.84
**Accuracy**	0.68	0.75	0.58	0.88 *	0.63	0.75	0.72	0.82 *
**Balanced Accuracy**	0.67	0.74	0.60	0.87 *	0.62	0.74	0.72	0.82 *

**Table 8:** Performance summary of classifying *Car* and *Pedestrian* events with LgR, MLP, SVM and RF classifier models using EEG and MI-based feature on the holdout test set among 738 observations where events due to pedestrian were considered as positive class. The number of observations with positive and negative class were 241 and 497 respectively. The highest accuracies obtained by using different feature sets are marked with (*).

Criteria	Using EEG-Based Features				Using MI-Based Features			
	LgR	MLP	SVM	RF	LgR	MLP	SVM	RF
**True Positive**	17	83	186	147	75	140	207	209
**False Negative**	224	158	55	94	166	101	34	32
**False Positive**	14	62	164	5	55	27	128	12
**True Negative**	483	435	333	492	442	470	369	485
**Sensitivity**	0.07	0.34	0.77	0.61	0.31	0.58	0.86	0.87
**Specificity**	0.97	0.88	0.67	0.99	0.89	0.95	0.74	0.98
**Precision**	0.55	0.57	0.53	0.97	0.58	0.84	0.62	0.95
**Recall**	0.07	0.34	0.77	0.61	0.31	0.58	0.86	0.87
$F_{1}$ **score**	0.13	0.43	0.63	0.75	0.40	0.69	0.72	0.90
**Accuracy**	0.68	0.70	0.70	0.87 *	0.70	0.83	0.78	0.94 *
**Balanced Accuracy**	0.52	0.61	0.72	0.80 *	0.60	0.76	0.80	0.92 *

## Abbreviations

The following abbreviations are used frequently in this manuscript:

EEG	Electroencephalography
IAF	Individual Alpha Frequency
LgR	Logistic Regression
LnR	Linear Regression
MI	Mutual Information
ML	Machine Learning
MLP	Multilayer Perceptron
MWL	Mental Workload
RF	Random Forest
SVM	Support Vector Machine
